# Evaluation of Anti-Inflammatory Activities of a Triterpene β-Elemonic Acid in Frankincense In Vivo and In Vitro

**DOI:** 10.3390/molecules24061187

**Published:** 2019-03-26

**Authors:** Yue Zhang, Ying-li Yu, Hua Tian, Ru-yu Bai, Ya-nan Bi, Xiao-mei Yuan, Li-kang Sun, Yan-ru Deng, Kun Zhou

**Affiliations:** 1 Institute of Traditional Chinese Medicine, Tianjin University of Traditional Chinese Medicine, 10 Poyang lake Road, Jinghai District, Tianjin 301617, China; zhyjoy1111@126.com (Y.Z.); yuguyu88@sina.com (Y.-l.Y.); tianhua2019@163.com (H.T.); 13337033276@163.com (R.-y.B.); bi15522880330@sina.com (Y.-n.B.); 13072003152@163.com (X.-m.Y.); likang.sun@bluewin.ch (L.-k.S.); 2 Tianjin Key Laboratory of Chinese medicine Pharmacology, 10 Poyang lake Road, Jinghai District, Tianjin 301617, China; 3 School of Chinese Materia Medica, Tianjin University of Traditional Chinese Medicine, 10 Poyang lake Road, Jinghai District, Tianjin 301617, China; 4 Ministry of Education Key Laboratory of Traditional Chinese Medical Formulae, Tianjin University of Traditional Chinese Medicine, 10 Poyang lake Road, Jinghai District, Tianjin 301617, China

**Keywords:** β-elemonic acid, anti-inflammatory, Frankincense, triterpene

## Abstract

The purpose of this research was to extract and separate the compounds from frankincense, and then evaluate their anti-inflammatory effects. The isolated compound was a representative tetracyclic triterpenes of glycine structure according to ^1^H-NMR and ^13^C-NMR spectra, which is β-elemonic acid (β-EA). We determined the content of six different localities of frankincense; the average content of β-EA was 41.96 mg/g. The toxic effects of β-EA administration (400, 200, 100 mg/kg) for four weeks in Kunming (KM) mice were observed. Compared with the control group, the body weight of mice, the visceral coefficients and serum indicators in the β-EA groups showed no systematic variations. The anti-inflammatory effects of β-EA were evaluated in LPS-induced RAW264.7 cells, xylene-induced induced ear inflammation in mice, carrageenin-induced paw edema in mice, and cotton pellet induced granuloma formation in rats. β-EA inhibited overproduction of tumor necrosis factor-α(TNF-α), interleukin-6 (IL-6), monocyte chemotactic protein 1 (MCP-1), soluble TNF receptor 1 (sTNF R1), Eotaxin-2, Interleukin 10 (IL-10) and granulocyte colony-stimulating factor (GCSF) in the RAW264.7 cells. Intragastric administration with β-EA (300, 200, and 100 mg/kg in mice, and 210, 140, and 70 mg/kg in rats) all produced distinct anti-inflammatory effects in vivo in a dose-dependent manner. Following treatment with β-EA (300 mg/kg, i.g.), the NO level in mice ears and PGE2 in mice paws both decreased (*p <* 0.01). In conclusion, our study indicates that β-EA could be a potential anti-inflammatory agent for the treatment of inflammatory diseases.

## 1. Introduction

Inflammation is the primary response to infection or injury to clear dead cells or agent and to promote tissue repair. However, inflammation can lead to tissue damage itself and dysfunction. It is a complex process regulated by a cascade of inflammatory mediators, including nitric oxide (NO); prostaglandin E2(PGE 2); cytokines, such as tumor necrosis factor-α (TNF-α) or interleukin-6 (IL-6),; etc. [1]. Recent studies have shown that inflammation was associated not only with wounds, trauma, and swelling [2], but also with many chronic diseases, such as cancer, arthritis, osteoporosis, asthma, Alzheimer’s disease, obesity, diabetes, and cardiovascular disease [3]. In conventional therapy, steroidal anti-inflammatory drugs and non-steroidal anti-inflammatory drugs (NSAIDs) are used to treat acute inflammation. Although very effective, their long-term use is associated with a broad spectrum of adverse reactions in the liver, kidney, cardiovascular system, skin, and gut [4,5]. Natural substances represent significant therapeutic potential on inflammation and usually have a wide safety range, thus could possibly be used for the design of innovative anti- inflammation drugs [6].

Frankincense (also identified as *Olibanum or Salai guggul*), the gum resin derived from *Boswellia* species, is widely used as an anti-inflammatory medicinal herb to treat conditions such as arthritis, asthma and inflammatory bowel disease [7,8]. 11-keto-β-boswellic acid (KBA) and 3-O-acetyl-11-keto-β-boswellic acid (AKBA) are the main active ingredients in frankincense. KBA is proposed to act as an inhibitor of 5-lipoxygenase (5-LO) [9] while AKBA is a natural inhibitor of the transcription factor NF-kB. In vitro and in vivo studies have demonstrated their anti-inflammatory effects [10,11]. Recently, other components, such as β-boswellic acid (βBA), have also been suggested as anti-inflammatory molecules [12]. However, to find other compounds in frankincense with anti-inflammatory effects still attracts interests of many researchers. This work extracted and separated compounds from frankincense, and then evaluated their toxicity in mice. Finally, we evaluated their effects on the inflammatory response, to establish the pharmacological basis for its continuous use.

## 2. Results

### 2.1. Structural Elucidation of the Isolated Compounds

**Figure 1:**
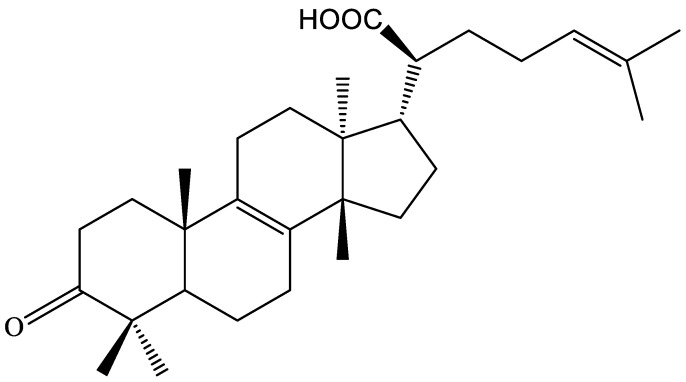
The chemical structure of the compound (β-elemonic acid, β-EA).

According to the result of Thin Layer Chromatography (TLC), only one compound was obtained (21.5 g) using 10% ethanol sulfate solution with color red. The identified compound was a colorless needle crystal. ^1^H-NMR spectrum of the compound revealed seven methyl groups corresponding to peaks at δ 0.82 (3H, s), 0.90 (3H, s), 1.03(3H, s), 1.05 (3H, s), 1.10 (3H, s), 1.59 (3H, s), and 1.68 (3H, s), which suggests that it may be a triterpenoid. ^1^H-NMR spectrum also showed peaks at δ1.59 and 1.68 that can be assigned to highly connected carbon atoms of C=C double bond. The peak at δ 5.10 (1H, t, *J* = 6.6 Hz) indicates presence of -CH2-CH=C(CH3). The peak at δ 7.27 (1H, s) can be assigned to carboxyl proton. The ^13^C-NMR spectra of the compound revealed that the compound possesses a total of 30 carbons atoms; the peaks were related to seven methyl groups. The peaks at δ 133.5 and 134.3 are assigned to C=C double bond carbons (C-8 and C-9, respectively) in the cyclic structure of the compound. The peaks at δ 123.4 and 132.2 were assigned to the external C=C double bond carbon atoms. The peaks at δ217.8 and 182.8 were assigned to ketone carbonyl and carbonyl carbon atom. Thus, based on the above spectral data, according to the literature [13], the compound was found to be identical with β-elemonic acid in its structure (β-EA, Figure 1)

### 2.2. The Content Determination of β-EA

The peaks were identified as β-EA using the analytical standard, at retention time (Rt) of 12.3 min. The calibration curve was obtained as the following:

*y* = 1.5803*x* + 7.6082, r^2^ = 0.9991.

The relative standard deviations (RSDs) for β-EA were 1.12% and 0.91% in repeated test, indicating a satisfactory precision and repeatability. The stability presented as RSD was 1.89%, indicating samples are stable within 24 h. The recovery was 99.24% for β-EA. The results indicate that the efficiency of sample preparation was acceptable in the current condition.

β-EA concentrations for each sample of six batches of frankincense from different origins, calculated based on the peak area and the calibration curve, were 40.65 (A), 38.79 (B), 45.59 (C), 43.11 (D), 43.26 (E), and 43.34 (F) mg/g.

### 2.3. Effect s of β-EA on Cell Viability and LPS Induced NO Production

**Figure 2:**
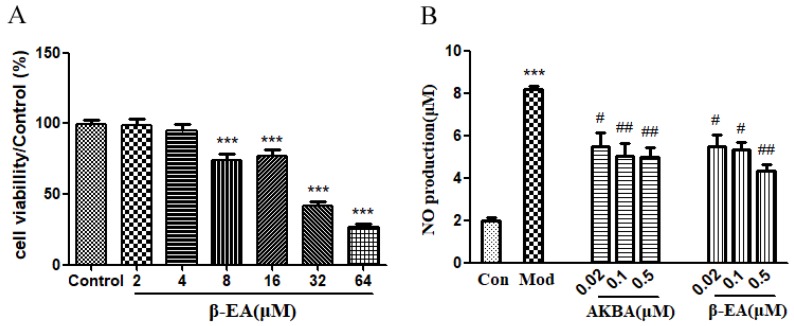
Effects of β-EA on cell viability and LPS induced NO production. RAW 264.7 cells were incubated with different concentrations of β-EA for 24 h. The cell viability was detected by MTT assay (**A**). Cells were treated with various concentrations of β-EA or AKBA for 24 h in the absence or presence of LPS. The NO production (**B**) was detected using a Griess assay kit. *** *p* < 0.001, significant difference from the control group; # *p* < 0.05, ## *p* < 0.01, significant difference from the LPS-treated group.

Overall, 8, 16, 32 and 64 μM of β-EA significantly reduced the cell viability to 74.3%,77.2%,41.6% and 26.7%, respectively (Figure 2A). However, the decrease disappeared or reduced when the concentration was 2 μM and 4 μM, indicating that β-EA has no cytotoxic effects on RAW 264.7 cells under the concentration of 4 μM.

The anti-inflammatory activities of β-EA and AKBA were then determined. As shown in Figure 2B, lipopolysaccharide (LPS) treatment (1 μg/mL) significantly increased the NO level in RAW 264.7 cells. Both β-EA and AKBA inhibited the NO production in concentration-dependent manners (all *p* < 0.05). Compared to the model group, the NO production in the AKBA and β-EA groups (0.02–0.5μM) were decreased, respectively.

### 2.4. Effect of β-EA on Inflammatory Factors In Vitro

**Figure 3:**
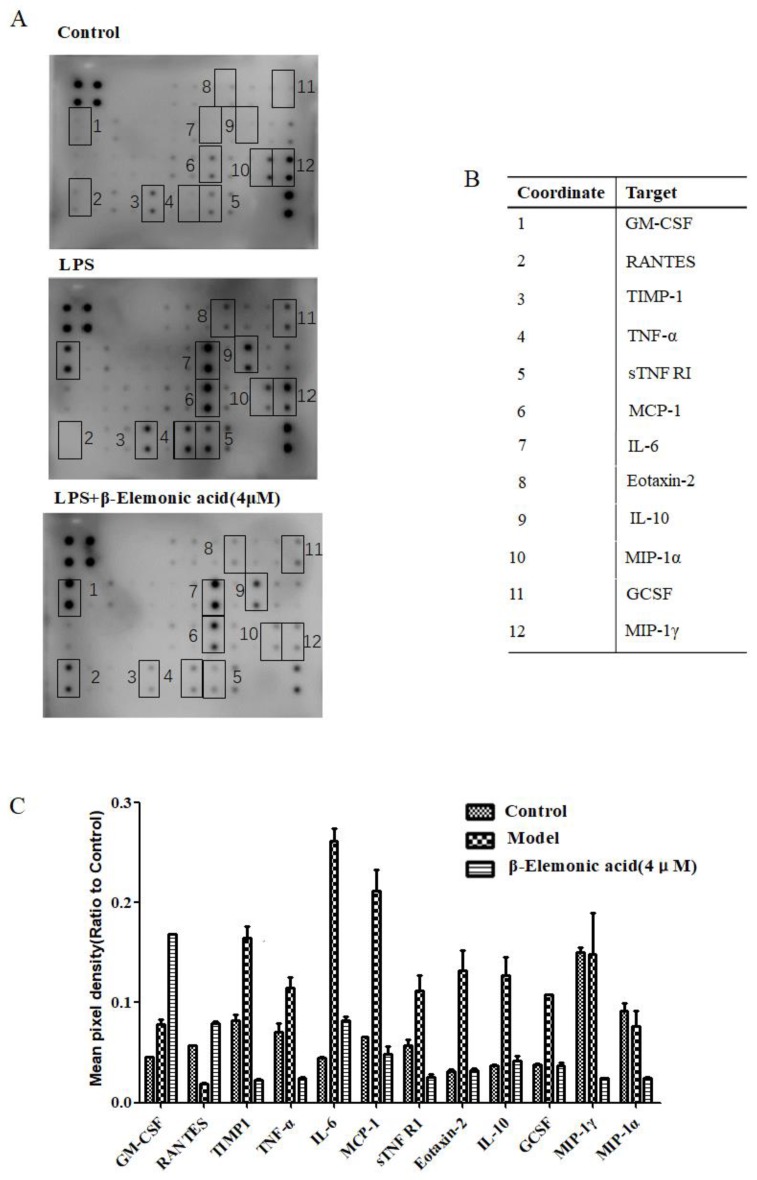
The effect of LPS treatment on the expression of inflammatory cytokines by RAW264.7 cells using antibody array membrane. (**A**) RAW264.7 cells were cultured in DMEM and divided into three treatment groups: control, LPS and LPS+ β-EA (4 μM). After 24 h of incubation, the cell supernatant were collected and incubated with the antibody array membrane. (**B**) Corresponding to the number in the A picture. (**C**) A visual comparison between the three arrays performed by Quantity One. Data were analyzed by densitometric analysis. Then, background was subtracted and results were normalized against the average of positive controls.

In our study, we used an antibody array membrane as a screening tool to assess the effect of inflammation and β-EA treatment on the expression of cytokines and chemokines in RAW264.7. A visual comparison between the three arrays performed by Quantity One. Software revealed that twelve cytokines were especially affected by the treatment: granulocyte-macrophage colony-stimulating factor (GM-CSF), regulated upon activation normal T-cell expressed and secreted (RANTES), Tissue inhibitor of metalloproteinase 1(TIMP1), tumor necrosis factor-α (TNF-α), Interleukin 6 (IL-6), monocyte chemotactic protein 1(MCP-1), soluble TNF receptor 1 (sTNF R1), Eotaxin-2, Interleukin 10 (IL-10), granulocyte colony-stimulating factor (GCSF) and macrophage inflammatory protein 1 alpha/gamma (MIP1α/γ). β-EA (4 μM) inhibited the overproduction of cytokines (TNF-α, IL-6, MCP-1, sTNF R1, Eotaxin-2, IL-10 and GCSF) induced by inflammation (Figure 3).

### 2.5. Short-Term Toxicity Test

After treatment with β-EA for four weeks, compared with the control group, the body weight of mice in the β-EA groups showed no systematic variations. Meanwhile. no significant changes were observed in liver and kidney visceral coefficients (Table 1). No significant changes were observed between β-EA groups and control group in serum indicators including alanine aminotransferase (ALT), aspartate aminotransferase (AST), alkaline phosphatase (ALP), total bilirubin (TBIL), triglyceride (TG), total cholesterol (TC), glucose (GLU), blood urea nitrogen (BUN), creatinine (CRE), creatine kinase (CK), total protein (TP), and albumin (ALB) (Table 2).

### 2.6. Effect s of β-EA on Acute Topical Edema

As shown in Table 3, the right ears of mice showed obvious edema after xylene administration with the swelling degree increasing to 20.75 ± 4.09. β-EA treatment (300, 200, and 100 mg/kg) significantly decreased the weight of inflamed ear in a dose-dependent manner. At the dose of 300 mg/kg (i.g.), β-EA inhibited the inflammation percentage value to 53.92% (*p* < 0.01), which is comparable to aspirin (63.66%, *p* < 0.01). Besides, the NO production in the inflamed ears was also detected. It was found that 300 mg/kg and 200 mg/kg of β-EA significantly decreased the NO production with an inhibition percentage of 65.07% and 46.44%, respectively.

### 2.7. Effects of β-EA on Acute Systemic Inflammation

Effects of β-EA on carrageenan-induced paw edema from 0.5 to 4 h after subcutaneous injection are shown in Table 4. The reduction of carrageenin-induced paw edema by β-EA was observed as early as 30 min. β-EA decreased the paw edema in a dose- and time-dependent manner (*p* < 0.05, *p* < 0.01). The maximum inhibition of inflammation was up to 41.31% at the dose of 300 mg/kg (i.g.), whereas aspirin showed 48.29% inhibition at the same time. Moreover, carrageenin significantly increased the PGE2 levels in the inflamed feet of mice and β-EA significantly decreased the level in a dose-dependent manner (Table 5, *p* < 0.05 or *p* < 0.01).

### 2.8. Effect of β-EA on Cotton Pellet Induced Granuloma Formation

**Figure 4:**
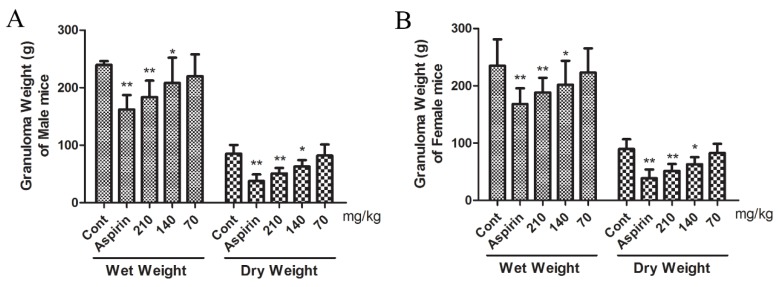
Effect of β-EA on Cotton Pellet Induced Granuloma Formation (*n* = 5). (**A**) Granuloma weight of male mice. (**B**) Granuloma weight of female mice. * p < 0.05, ** p < 0.01, significant difference from the control group.

The granuloma tissue formation is a feature of chronic inflammation. As shown in Figure 4, subcutaneous cotton pellet implantation produced granuloma. β-EA (140 and 210 mg/kg) or aspirin (300 mg/kg) exhibited significant inhibition of both wet and dry granuloma formation in male and female rats. It was found that 210 mg/kg of β-EA reduced dry granuloma from 89.65 ± 16.87 to 51.33 ± 12.16 (*p* < 0.01) in female rats and from 85.16 ± 15.28 to 50.81 ± 9.46 (*p* < 0.01) in male rats. In addition, 140 mg/kg of β-EA reduced dry granuloma to 63.05 ± 12.37 (*p* < 0.05) in female rats and 62.84 ± 11.31 (*p* < 0.05) in male rats, respectively. The reduction was significant (*p* < 0.01 or *p* < 0.05) when compared to control and the effect was slightly lower than aspirin.

## 3. Discussion

Inflammation is widely involved in the development of many systemic diseases. Recently, increasing attention has been paid to the development of new therapeutic agents from natural products or traditional medicinal plants due to their wider safety range. Several natural products have been comprehensively explored as a source of therapeutic agents [14]. Frankincense, a widely used herbal medicine, has been found to have anti-inflammatory and anti-cancer effects [15,16]; however, most of the previous studies only focus on the effect on the crude extract of frankincense or some of the compounds in frankincense such as boswellic acids (BAs), AKBA or KBA, while β-EA is under researched. This study purified a triterpene from frankincense and demonstrated its anti-inflammatory activities. Our results demonstrate that β-EA derived from frankincense exhibited potent analgesic and anti-inflammatory activities.

β-EA is a known triterpene [17]. Few studies have investigated its pharmaceutical effects [18] and it has not yet been used in clinic alone. In this research, we isolated β-EA from frankincense and determined the content of six different localities of frankincense. It was shown that β-EA is rich in frankincense with the average content of 41.96 mg/g.

Since β-EA at concentration over > 4 μM displayed significant cytotoxicity in RAW 264.7 cells, only lower dosage of β-EA administration (100, 200, 400 mg/kg, i.g.) were tested for their toxic effect in Kunming mice (KM). After treatment with β-EA for four weeks, compared with the control group, the body weight of mice, the visceral coefficients and serum indicators in the β-EA groups showed no systematic variations. These results indicate that β-EA has no obvious toxicity in vivo.

Next, we studied the efficacy of β-EA in vitro and in vivo. The anti-inflammatory effect of β-EA was found in vitro. We used an antibody array membrane to explore the effect of β-EA treatment on expressions of cytokines and chemokines in RAW264.7. In total, 40 cytokines were screened and 12 cytokines change obviously after β-EA treatment. Among these, TNF-α, IL-6, MCP-1, sTNF R1, Eotaxin-2, IL-10 and GCSF are important inflammatory mediators [19,20,21]. It is reported that pro-inflammatory cytokines such as TNF-α lead to an increased expression of PI3K and cause various pathological processes. Inflammasome adaptor molecules such as the apoptosis-associated speck–like protein, promote the development of inflammatory by increasing IL-18 and IL1-β levels [22]. AKBA, KBA and β-BA have an anti-inflammatory effect in vitro with IC_50_ values of 1.5–50 µM depending on the experimental settings [23,24,25]. β-EA (4 μM) inhibited the overproduction of TNF-α, IL-6, MCP-1, sTNF R1, Eotaxin-2, IL-10 and GCSF, suggesting that it has anti-inflammatory activity. After we isolated and extracted β-EA from frankincense, we compared the bioactivity of AKBA, which is the main active component of frankincense, in LPS-induced RAW264.7 cells. Effects of AKBA and β-EA on LPS induced NO production are shown in Figure 2B. Both β-EA and AKBA inhibited the NO production in concentration-dependent manners (*p* < 0.05), which confirmed the results of antibody array experimental data. β-EA showed good anti-inflammatory effect in vitro.

Then, animal experiments were conducted to verify its anti-inflammatory activity. Xylene-induced ear edema and carrageenin-induced rat paw edema are the classic models for inflammation tests [26,27]. Xylene induces instant irritation of the mouse ear, leading to fluid accumulation and edema characteristic of the acute inflammatory response. In xylene-induced ear edema study, 300 mg/kg β-EA could significantly relieve ear edema and the inhibition rate was equal to that in the aspirin-treated group. In addition, NO is a pro-inflammatory molecule; decreasing NO production is applied to treat inflammatory diseases [28]. The contents of NO in the β-EA treated groups were significantly decreased. Suppression of this response indicates that β-EA has topical anti-inflammatory activity. The model of carrageenan-induced inflammation has a significant predictive value for anti-inflammatory agents [29]. The process of carrageenan-induced inflammatory in the rat include three phages with several mediators released in sequence [30]. Histamine and 5-HT were released during the first hour, causing increased vascular permeability, which was maintained by released kinin for up to 2.5 h. After 2.5 h, the released mediator appeared to be a prostaglandin [31]. Administration of 300 mg/kg β-EA suppressed carrageenan-induced paw edema from 0.5 to 4 h post-injection. β-EA exerts effects in the first, second and third phase, which suggested its mechanism of anti-inflammation is related to both bradykinin and prostaglandins synthesis inhibition, as confirmed by the PGE2 levels in the inflamed feet of mice. β-EA significantly decreased the level of PGE2 in a dose-dependent manner (*p* < 0.05 or *p* < 0.01).

The inflammatory granuloma is a typical feature of established chronic inflammatory [32]. The formation of granulomatous tissue is identified using the dry weight of a cotton pellet [33]. During the repair process of inflammation, there is proliferation of neutrophils, macrophages, fibroblasts, and the multiplication of small blood vessels, which are the basic sources of highly vascularized reddish mass, termed granulation tissue [34]. Granulomas form relatively slowly over a period of days and are an important defense mechanism. Inflammatory mediators such as interleukin-1α (IL-1α), TNF-α and IL-6 [1] are recognized to be involved in the formation of the granuloma. In our studies, 210 mg/kg β-EA exhibited significant inhibitory activity of granuloma tissue formation in both male and female mice, which indicated the ability of β-EA to reduce the number of fibroblasts, synthesize of collagen and mucopolysaccharide, and exert good anti-inflammatory effect.

In conclusion, we isolated and characterized β-EA from frankincense. We determined the content of six different localities of frankincense with an average of 41.96 mg/g of β-EA. We also tested the toxic effect of lower dosage of β-EA administration in KM, and there was no apparent toxicity in mice after treatment with β-EA for four weeks. Present experimental results reveal that β-EA exhibited considerable anti-inflammatory activity in different inflammation experiments in LPS-induced RAW264.7 cells and in animal models. It provides a rationale for the use of β-EA in the therapy of inflammatory diseases.

## 4. Materials and Methods

### 4.1. Reagents

Frankincense was obtained from Anguo (Hebei, China). β-EA and AKBA were purchased from Chengdu Pufei De Biotech Co.,Ltd. (Chengdu, China). Aspirin was purchased from Bayer Healthcare Company Ltd. (Leverkusen, Germany). Carrageenan and xylene were obtained from Tianjin Jinbei Fine Chemical Co., Ltd. (Tianjin, China). Carboxymethylcellulose (CMC) was purchased from Concord Technology Co., Ltd. (TianJin, China). ELISA kits for NO and PGE-2 were obtained from Westang Co., Ltd. (Shanghai, China). Antibody array membrane was obtained from R&D Systems, Inc. (Minneapolis, USA). The AST, ALT, ALP, TC, TG, CRE, GLU, BUN, TBIL, CK, TP and ALB kits were purchased from BioSino Bio-technology and Science Inc. (Beijing, China).

### 4.2. Isolation and Characterization of Compounds

The frankincense (3 kg) was crushed with a material–liquid ratio of 1:8 with 95% ethanol reflux and extracted for 2 hthree times. The extracts were combined (2034.8 g) and then concentrated under vacuum. The extract was dissolved in petroleum ether and extracted with water to obtain petroleum ether layer extract (983 g). Then, part of the petroleum ether layer was taken out (500 g), and subjected to solvent-guided fractionation in a silica gel (100–200 mesh size). A column was successively eluted with petroleum ether and ethyl acetate (6:1). Every 400 mL eluent is a fraction. A total of 25 fractions were collected and the solvent was removed under reduced pressure using rotavapor (under reduced pressure). The developed spots on TLC plates were visualized under UV light at 254 and 365 nm and then by exposure to iodine chamber. The fractions that showed the same TLC development profiles (color and Rf) were combined and concentrated to dryness under reduced pressure using rotary evaporator. The structures of the compounds were elucidated based on combined spectral data, which include infrared, nuclear magnetic resonance (^1^H-NMR, ^13^C-NMR) spectra data and melting point values as well as comparison of these data with reported data in literature. The process can be seen in Figure S1.

### 4.3. Determination of β-EA in Frankincense of Different Origins by HPLC-ELSD

A HPLC system (Agilent 1260, USA) along with evaporation light scattering detector (ELSD, 2421) was used for the analysis. The compound was separated on WondaSil C18 column (4.6 mm × 150 mm, 5 µm) maintained at 35 °C with the mobile phase consisted of acetonitrile (A)and 0.05% formic acid (B). The flow rate was 1.0 mL/min. The gradient elution was as follows: 0–5 min, 85% A; 5–10 min, 90% A; 10–15 min, 90% A; 15–18 min, 90–93% A; 18–20 min, 93% A; 20–25 min, 93–100% A. The injection volume was 10 μL. The atomizer temperature was 35 °C, the drift tube temperature was 110 °C, pressure was 0.16 MPa, and the carrier gas was nitrogen. A standard stock solution was prepared by dissolving 10 mg of β-EA analytical standard in methanol (10 mL). The calibration curve was obtained by analyzing six serial dilutions (1.0, 0.8, 0.6, 0.4, 0.2 and 0.1 mg/mL) of the stock solution. The standard curve of β-EA was obtained by taking the natural logarithm of the control sample as the abscissa. The method was evaluated by intraday and interday variability. The RSDs were calculated as the measure of precision. In the repeatability examination, six replicates of the samples from the same batch were extracted and analyzed. To evaluate the stability of analytes, sample solutions were stored at room temperature and then analyzed by replicate injection at 0, 2, 4, 8, 12, 16, 20 and 24 h. The RSDs were used to assess the stability. The recovery was evaluated by adding the standard solution to samples, which was used to further investigate the accuracy of the method.

First, 0.1 g powder of six batches of frankincense from different origins (A–F) was weighed. The six batches of frankincense were samples from different origins identified by Qiduo Zhao (assistant professor, Tianjin University of Traditional Chinese Medicine), and the specimens reference numbers can be seen in Table 6. Then, 20 mL methanol was added and ultrasonic treatment was conducted. The solvent was recorded to dry, the residue was dissolved in methanol and transferred to a 10 mL volumetric flask. Methanol was added to scale. After shaking well, it was filtered with 0.22 μm microporous membrane. The β-EA peaks in the samples were identified based on the retention time on the chromatogram. All measurements were performed in triplicate and data were reported as mean ± SD.

### 4.4. Cell Viability Assay

Raw 264.7 murine macrophage cells were purchased from the Chinese Academy of Sciences (Shanghai, China). The cells were cultured at 37 °C in a 5% CO_2_ atmosphere with DMEM supplemented with 10% FBS and antibiotics (100 U/mL penicillin and 100 μg/mL streptomycin).

Cytotoxicity induced by β-EA were detected by MTT assay. RAW 264.7 cells (7.5 × 10^4^/well) were plated in 96-well plates overnight, and treated with various concentrations (2 μM, 4 μM, 8 μM, 16 μM, 32 μM and 64 μM) of β-EA for 24 h. MTT solution (5 mg/mL) was added to each well and plates were incubated for 4 h in a CO_2_ incubator at 37 °C. The absorbance was measured at 570 nm after the formazan crystals dissolved in 100 μL DMSO.

### 4.5. Measurement of Extracellular NO Production

The NO production induced by LPS was detected using a Griess assay kit (Beyotime Biotechnology, Shanghai, China) according to the manufacturer’s instructions. Briefly, RAW 264.7 cells (7.5 × 10^4^/well) were plated in 96-well plates overnight and treated with various concentrations (0.02 μM, 0.1 μM, and 0.5 μM) of β-EA or AKBA for 24 h in the absence or presence of LPS (1 μg/mL). Equal volumes (50 μL) of cell culture supernatant and Griess reagent (1:1 mixture of 1% sulfanilamide in 5% phosphoric acid and 0.1% N-(1-naphthyl) ethylene diamide dihydrocholide) were mixed and incubated at room temperature for 15 min. Absorbance was determined at 540 nm and the concentration of total nitrite + nitrate was calculated according to a standard curve of known nitrite concentrations.

### 4.6. Expression of Inflammatory Cytokines Using Antibody Array Membrane

The cells were seeded in 24-well plates (3 × 10^5^ cells/mL). The control group, model group (with LPS only), and the administration group were incubated with LPS and β-EA (4 μM) for 24 h at 37 °C. Cell-free supernatants were collected and stored at −20 °C until assayed for cytokines. To evaluate differential cytokine expression in the treated samples, antibody array membranes were used according to the manufacturer’s instructions. Briefly, the membranes were placed in the 8-well tray provided in the kit, and then 1 mL of each sample was incubated overnight into the designated well at 4 °C. Twenty-four hours later, the samples were aspirated and the membranes were washed with wash buffer and then incubated with 1 mL of biotin-conjugated anti-cytokines overnight at 4 °C. Then, the biotin-conjugated anti-cytokines were aspirated and washed again as previously described. Next, 2 mL of horseradish peroxidase-conjugated streptavidin was pipetted into each well and incubated overnight at 4 °C. Then, it was aspirated and the membranes were washed again by the previously described method. Detection buffer was used to develop a chemiluminescent signal and the C-DIGIT blot scanner was used to detect the signal intensity. Measurements of the intensity of signals on the array membrane were performed using Quantity One software. Data were normalized to the positive control signals and the relative cytokine expression was determined.

### 4.7. Animals

Male Kunming (KM) mice (18–22 g), male and female Sprague-Dawley (SD) rats (220–240 g) were obtained from the pathogen-free facility at Beijing HFK Bioscience Technology Co. Ltd. (Beijing, China). The animals were allowed to acclimatize to the laboratory environment for 7 days prior to the experiment. They were kept in plastic cages at 24 ± 2 °C with free access to pellet food and water and on a 12 h light/dark cycle. The animal experiments were approved by the Laboratory Animal Ethics Committee of Tianjin University of Traditional Chinese Medicine (permit number: TCMLAEC 2014101).

### 4.8. Short-Term Toxicity Test

A total of 40 mice were randomly divided into four groups (*n* = 10) to receive 0.5% CMCNa (the vehicle control group), or 100, 200 or 400 mg/kg β-EA solution with orally administered for 4 weeks. The general behavior of the mice was observed and recorded daily, and the weight of the mice were detected weekly. Finally, the mice were anesthetized and then euthanized. Blood was collected to examine serum biochemical parameters such as ALT, TC, TG, GLU, AST, ALP, TBIL, BUN, CRE, CK, TP and ALB, and the liver and kidney were tested for visceral coefficients.

### 4.9. Xylene-Induced Ear Edema

Fifty male mice were randomly divided into five groups (*n* = 10) to receive 0.5% CMCNa (the vehicle control group), 500 mg/kg aspirin (the positive group), or 100, 200 or 300 mg/kg β-EA solution, which was orally administered for 7 days (0.04 mL/g/day). An hour after the last drug administration, 30 μL of xylene was applied to the surface of the mouse right ear for 30 min. Then, the whole blood was collected from the mice orbit and both ears were removed. Circular sections were punched out of the right (treated) and left (untreated) ear lobes using a cork borer (8 mm diameter) and weighed. Ear edema was expressed as the difference value of the right (Vr) and left ears (Vl). The anti-inflammatory activity was evaluated as percent edema inhibition in the treated animals relative to control animals using the relation:

Inhibition of edema (%) = ((Vr − Vl)control − (Vr − Vl)treated)/(Vr − Vl)control × 100%

The whole blood was placed on ice, coagulated, and centrifuged at 3000 rpm for 10 min to collect serum for the following determine of NO by the Enzyme-linked immunosorbent assay (ELISA) according to the manufacturer’s instructions.

### 4.10. Carrageenan-Induced Rat Paw Edema

Another 50 male mice were randomly divided into five groups (*n* = 10), administered by 0.5% CMCNa (the vehicle control group), 500 mg/kg aspirin (the positive group), or 100, 200 or 300 mg/kg β-EA for 7 days as described above. Thirty minutes after the last drug administration, acute inflammation was induced by injecting 50 μL of 0.1% (*w/v*) carrageenan into the sub-plantar region of the right hind paw of each mouse. The paw volume was measured before and at 5 min, 30 min, 1 h, 2 h and 4 h after the injection of carrageenan. Feet edema was expressed as the difference value of the right (Pr) and left feet (Pl). Anti-inflammatory activity was evaluated as percent edema inhibition in the treated animals relative to control animals using the relation:

Edema degree = Pf − P0

The percentage inhibition of feet swelling = ((Pr − Pl)control − (Pr − Pl)treated)/(Pr − Pl)control × 100%

After the last measurement, all mice were anesthetized with ether and cut the feet. The cut feet were weighed, chopped into pieces, and soaked in 2 mL of normal saline solution for 1.5 h. After centrifugated at 3000 rpm for 10 min, 0.25 mL of the supernatant fraction was collected and inspected by the ELISA assays of PGE2.

### 4.11. Cotton Pellet Induced Granuloma Formation

The effect of β-EA on granulomatous inflammation was evaluated using the cotton pellet granuloma test with modification. Twenty-five male and 25 female SD rats were randomly divided into 5 groups (*n* = 5), respectively, according to their weights. The sterile autoclaved cotton pellets (20 mg) were implanted on each side of the subcutaneous dead space in the depilated axial region of rats under chloral hydrate anesthesia. The wounds were sutured with silk and the animals were placed in their individual cages to receive oral administrations of 0.5% CMCNa (the vehicle control group), 300 mg/kg aspirin (the positive group), or 70, 140 or 210 mg/kg β-EA, respectively. Treatment was given once daily via the oral route (0.3 mL/100g/day) for 14 days. After the last administration, the animals were sacrificed by overdose of chloroform anesthesia and the pellets with the granuloma tissues formed were carefully excised, dried overnight in a hot air oven at 70 °C to a constant weight. The weight of granuloma tissue formed in treated rats was compared to control.

### 4.12. Statistical Analysis

Results are presented as mean ± SEM. Data obtained were analyzed using One-Way ANOVA. Differences between means of treated and control groups were accepted as significant at *p < 0.05*.

## Figures and Tables

**Table 1:** Effects of repeated administration of β-EA on visceral coefficients in mice treated for four weeks (mean ± SD, *n* = 10).

	Dose (mg/kg, i.g.)	Body Weight (g)	Liver (g)	Liver Coefficient	Kidney (g)	Kidney Coefficient
Control	-	39.16 ± 3.35	1.60 ± 0.21	4.08 ± 0.22	0.54 ± 0.12	13.26 ± 2.60
β-EA	400	39.02 ± 4.57	1.56 ± 0.21	3.98 ± 0.28	0.51 ± 0.13	12.95 ± 3.44
β-EA	200	38.60 ± 2.85	1.55 ± 0.21	4.02 ± 0.32	0.50 ± 0.11	12.53 ± 2.16
β-EA	100	40.37 ± 3.67	1.59 ± 0.20	3.94 ± 0.40	0.53 ± 0.09	13.59 ± 2.71

**Table 2:** Effects of repeated administration of β-EA on blood biochemical parameters in mice treated for 4 weeks (mean ± SD, *n* = 10).

Parameter	Control	400 mg/kg	200 mg/kg	100 mg/kg
ALT	30.29 ± 4.80	27.56 ± 4.83	27.70 ± 7.92	28.86 ± 5.79
AST	132.3 ± 15.9	132.7 ± 25.7	134.50 ±23.5	136.6 ± 28.3
ALP	109.3 ± 41.8	119.7 ± 43.6	126.9 ± 27.2	115.3 ± 22.5
TBIL	0.24 ± 0.04	0.23 ± 0.05	0.22 ± 0.04	0.22 ± 0.05
TG	0.73 ± 0.31	0.67 ± 0.16	0.71 ± 0.16	0.77 ± 0.07
TC	2.27 ± 0.31	2.21 ± 0.44	2.03 ± 0.29	2.11 ± 0.19
GLU	5.37 ± 0.80	5.04 ± 1.10	5.31 ± 0.85	5.95 ± 1.11
BUN	6.33 ± 0.31	6.17 ± 1.20	6.31 ± 1.88	7.66 ± 1.99
CRE	36.04 ± 3.31	33.94 ± 4.43	37.545.39	39.64 ± 5.13
CK	639.1 ± 219.2	708.3 ± 240.9	596.2 ± 306.9	785.7 ± 270.9
TP	57.19 ± 2.61	54.63 ± 2.69	56.19 ± 3.35	55.89 ± 3.55
ALB	23.26 ± 1.46	23.23 ± 1.37	23.88 ± 1.40	23.69 ± 1.90

**Table 3:** Effect of β-EA on xylene induced ear inflammation in mice (*n* = 10).

	Dose (mg/kg)	Left Ear (mg)	Right Ear (mg)	Topical Edema (mg)	Inhibition (%)	OD	NO	Inhibition
								(%)
Control	-	15.12 ± 3.12	35.87 ± 5.13	20.75 ± 4.09	-	1.402 ± 0.02	39.133 ± 7.68	-
Aspirin	500	15.24 ± 3.20	22.78 ± 5.13	7.54 ± 1.21**	63.66	0.655 ± 0.06	11.451 ± 2.31**	70.73
β-EA	300	15.13 ± 4.14	24.69 ± 4.18	9.56 ± 1.62**	53.92	0.715 ± 0.03	13.668 ± 2.11**	65.07
β-EA	200	15.39 ± 3.50	28.06 ± 3.16	12.67 ± 1.90*	38.31	1.002 ± 0.11	21.001 ± 5.08 *	46.44
β-EA	100	14.98 ± 3.21	36.07 ± 6.10	21.09 ± 3.23	−1.6	1.145 ± 0.13	39.624 ± 8.05	16.68

* p < 0.05, ** p < 0.01, significant difference from the control group

**Table 4:** Effect of β-EA on carrageenin-induced paw edema in mice (*n* = 10).

	Dose (mg/kg, i.g.)	Swelling Degree (mm)					
		0 min	5 min	30 min	1 h	2 h	4 h
Control	-	2.65 ± 0.12	1.65 ± 0.23	1.60 ± 0.17	1.62 ± 0.16	1.58 ± 0.16	1.60 ± 0.11
Aspirin	500	2.64 ± 0.20	1.56 ± 0.21	0.81 ± 0.09	0.49 ± 0.03 **	0.46 ± 0.19 **	0.47 ± 0.01 **
β-EA	300	2.65 ± 0.18	1.57 ± 0.13	0.86 ± 0.15 *	0.66 ± 0.14 **	0.63 ± 0.15 **	0.64 ± 0.04 **
β-EA	200	2.64 ± 0.15	1.55 ± 0.24	1.43 ± 0.08	0.79 ± 0.11 *	0.76 ± 0.14 *	0.71 ± 0.17 *
β-EA	100	2.64 ± 0.09	1.56 ± 0.25	1.51 ± 0.20	1.49 ± 0.10	1.48 ± 0.13	1.50 ± 0.06

* p < 0.05, ** p < 0.01, significant difference from the control group.

**Table 5:** Effect of the biflavonoid on carrageenin-induced paw edema in rats (*n* = 10).

	Dose (mg/kg, i.g.)	Left Feet (mg)	Right Feet (mg)	Paw Oedema (mg)	Inhibition (%)	PGE2 (OD/Weights)
Control	-	17.12 ± 3.21	71.87 ± 15.14	54.75 ± 8.89	-	1.25 ± 0.18
Aspirin	500	17.24 ± 3.18	44.03 ± 13.18	26.79 ± 6.17 **	48.29	0.36 ± 0.07 **
β-EA	300	17.53 ± 5.16	47.89 ± 14.11	30.36 ± 5.21 **	41.31	0.65 ± 0.21 **
β-EA	200	17.39 ± 3.19	51.90 ± 12.15	34.51 ± 4.58 *	33.31	0.86 ± 0.26 *
β-EA	100	17.62 ± 4.11	58.71 ± 14.82	41.09 ± 6.99	20.59	1.13 ± 0.05

* p < 0.05, ** p < 0.01, significant difference from the control group.

**Table 6:** Six batches of frankincense samples.

Origin code	Source of Purchase	Specimens Reference Numbers
A	Gansu Provincial Hospital	RX2016052301
B	Taiyuan Central Hospital	RX2016052302
C	Jincheng General Hospital	RX2016052303
D	Hanchuan Hospital of Traditional Chinese Medicine	RX2016052304
E	Tianjin Lerentang	RX2016052305
F	Tianjin Tongrentang	RX2016052306

## Supplementary Materials

The following are available online. Figure S1. Isolation of the compound from frankincense.
