# Supplementary material for: Evaluation of Anti-Inflammatory Activities of a Triterpene β-Elemonic Acid in Frankincense In Vivo and In Vitro
Source: Molecules. 2019 Mar 26;24(6):1187. doi: 10.3390/molecules24061187 (PMC6471661; doi:10.3390/molecules24061187)
Supplement: Supplementary file 1 [file molecules-24-01187-s001.zip › molecules-465520 proof supple/molecules-465520 revised supple2/Supplementary Material/Figure S1.pdf]

Evaluation of anti-inflammatory activities of a triterpene β-elemonic acid in Frankincense *in vivo* and *in vitro*

Yue Zhang[1,2†], Ying-li Yu[1,2†], Hua Tian[1], Ru-yu Bai[1], Ya-nan Bi[1], Xiao-mei Yuan[1], Li-kang Sun[1,2], Yan-ru Deng[3*], Kun Zhou[1,2,4*]

1 Institute of Traditional Chinese Medicine, Tianjin University of Traditional Chinese Medicine, 10 Poyang lake Road, Jinghai District, Tianjin 301617, China, z.k.ken@263.net(K. Zhou)
2 Tianjin Key Laboratory of Chinese medicine Pharmacology, 10 Poyang lake Road, Jinghai District, Tianjin 301617, China, z.k.ken@263.net (K. Zhou)
3 School of Chinese Materia Medica, Tianjin University of Traditional Chinese Medicine, 10 Poyang lake Road, Jinghai District, Tianjin 301617, China, dyanru@sina.com (Y.R. Deng)
4 Ministry of Education Key Laboratory of Traditional Chinese Medical Formulae, Tianjin University of Traditional Chinese Medicine, 10 Poyang lake Road, Jinghai District, Tianjin 301617, China, z.k.ken@263.net(K. Zhou)

† These authors contributed equally to this work.* Correspondence: z.k.ken@263.net; Tel./Fax: +86-22-5959-6163(K. Zhou); dyanru@sina.com; Tel./Fax: +86-22-5959-6225 (Y.R. Deng)

Supplementary Materials:

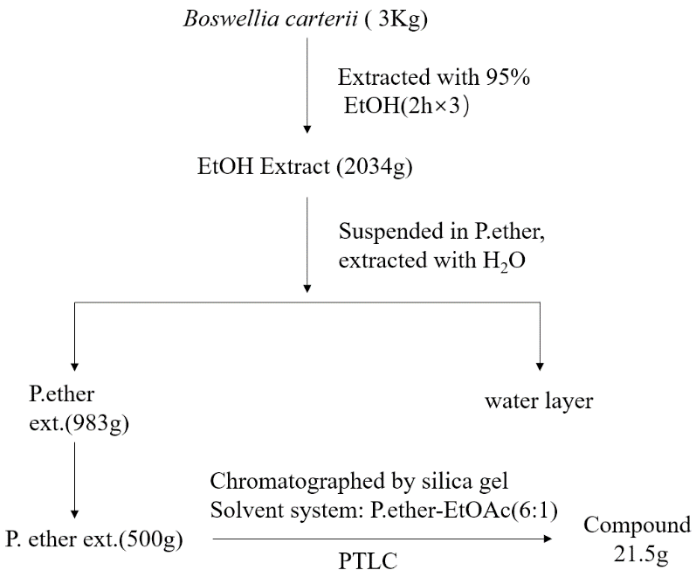

Figure S1. Isolation of the compound from frankincense.
