# Supplementary figures and images for: Evaluation of Anti-Inflammatory Activities of a Triterpene β-Elemonic Acid in Frankincense In Vivo and In Vitro
Source: Molecules. 2019 Mar 26;24(6):1187. doi: 10.3390/molecules24061187 (PMC6471661; doi:10.3390/molecules24061187)

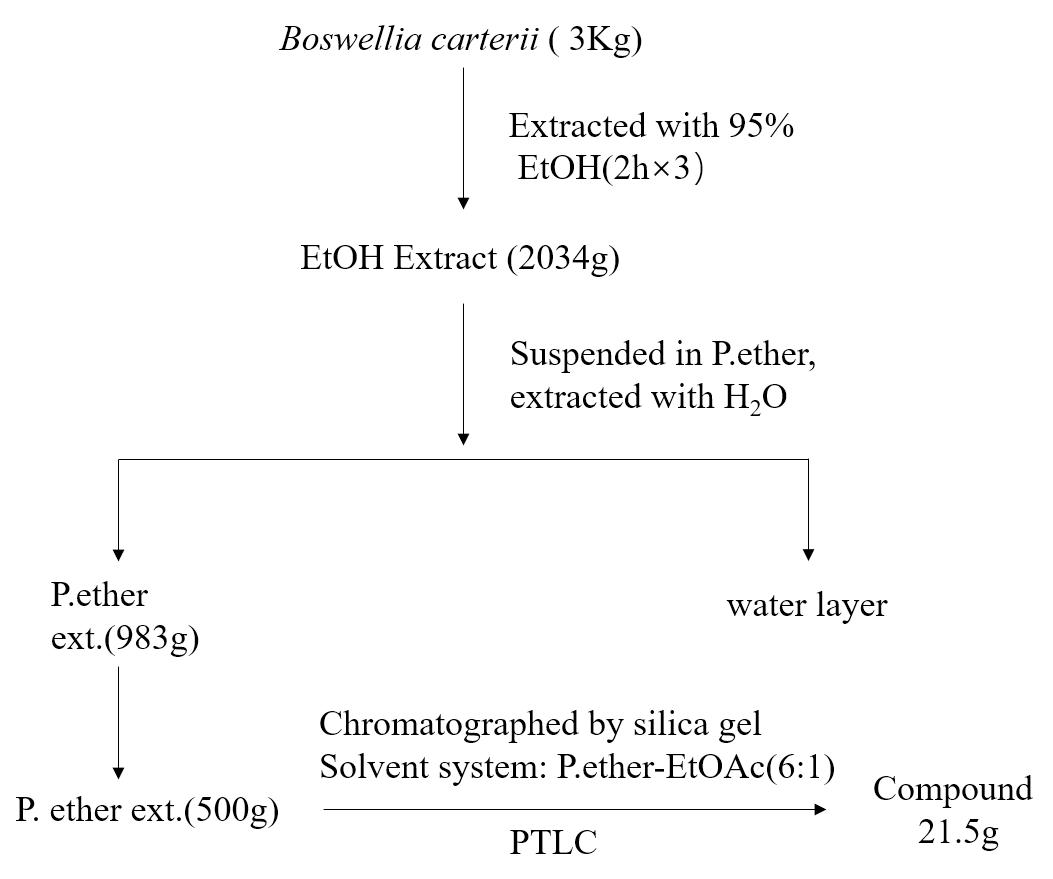

Supplement: Supplementary file 1 [file molecules-24-01187-s001.zip › molecules-465520 proof supple/molecules-465520 revised supple2/Supplementary Material/figure S1.tif]
